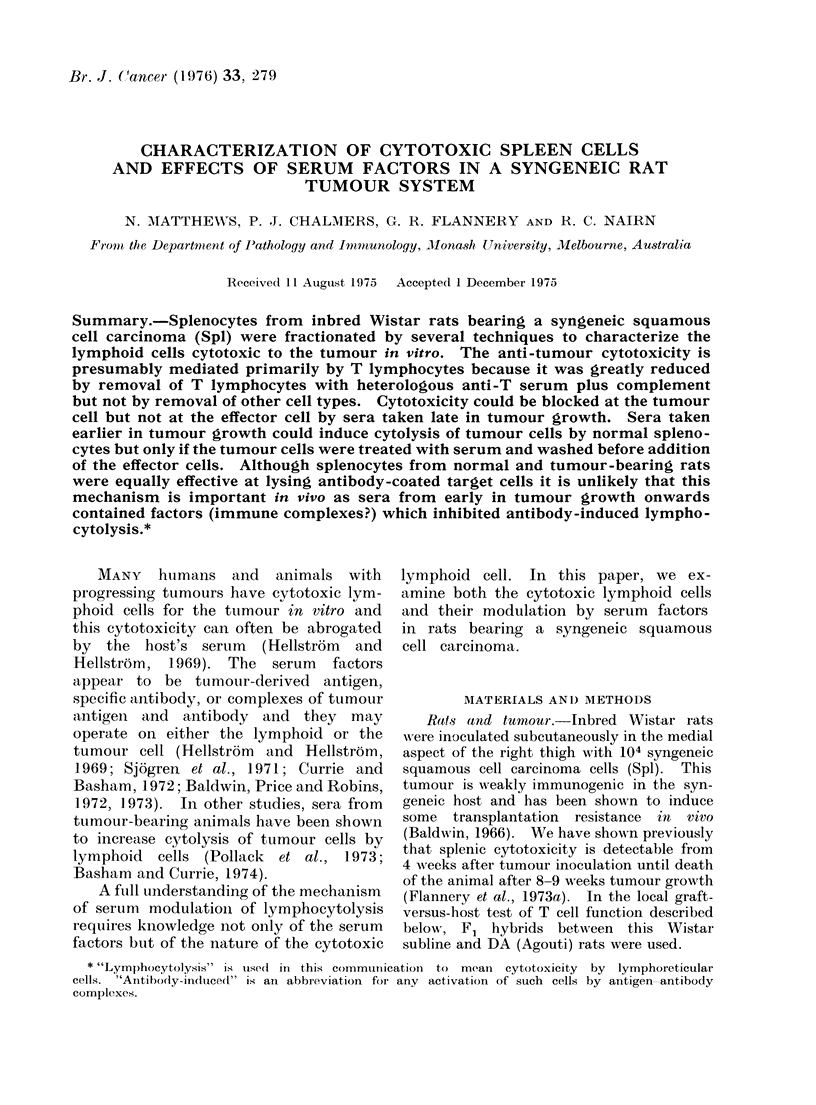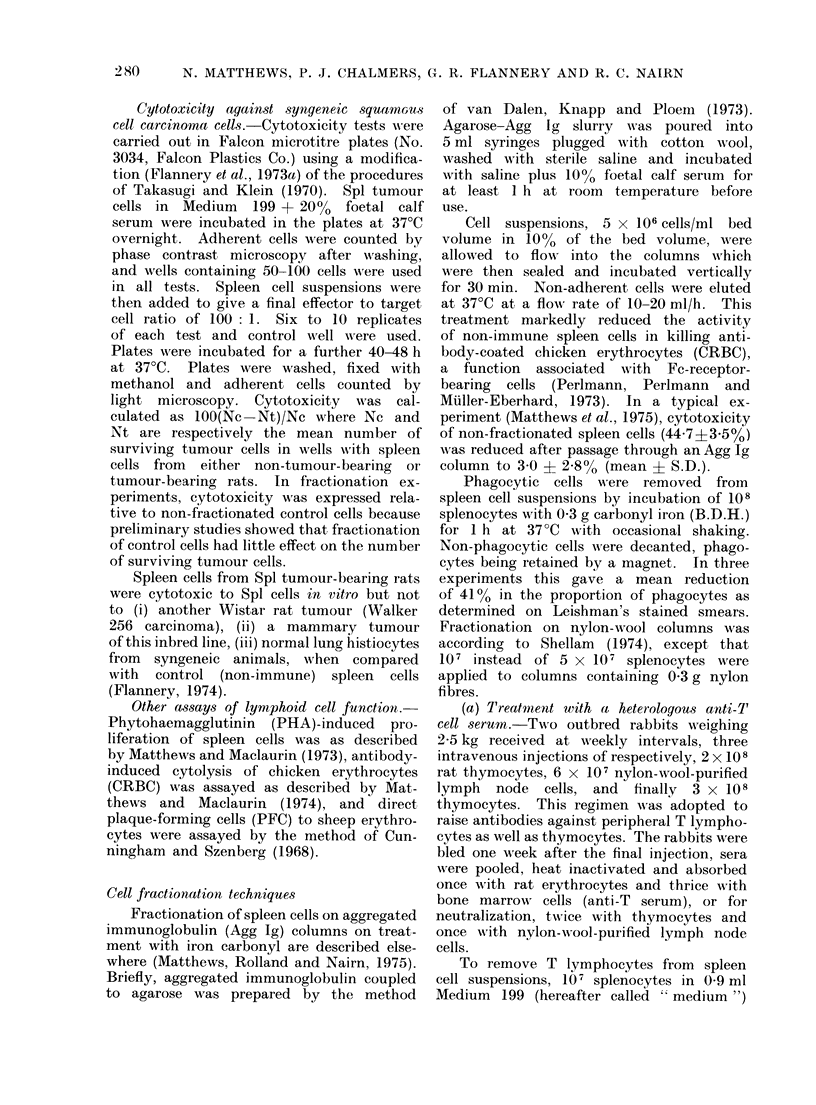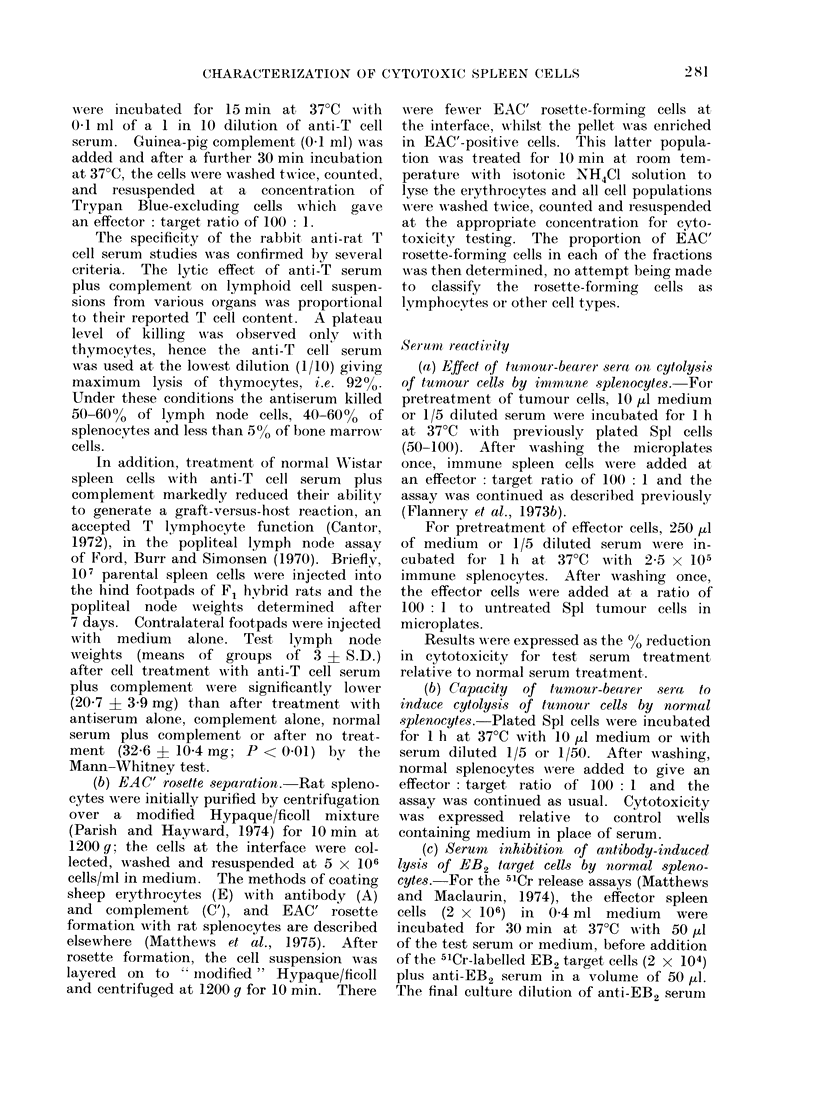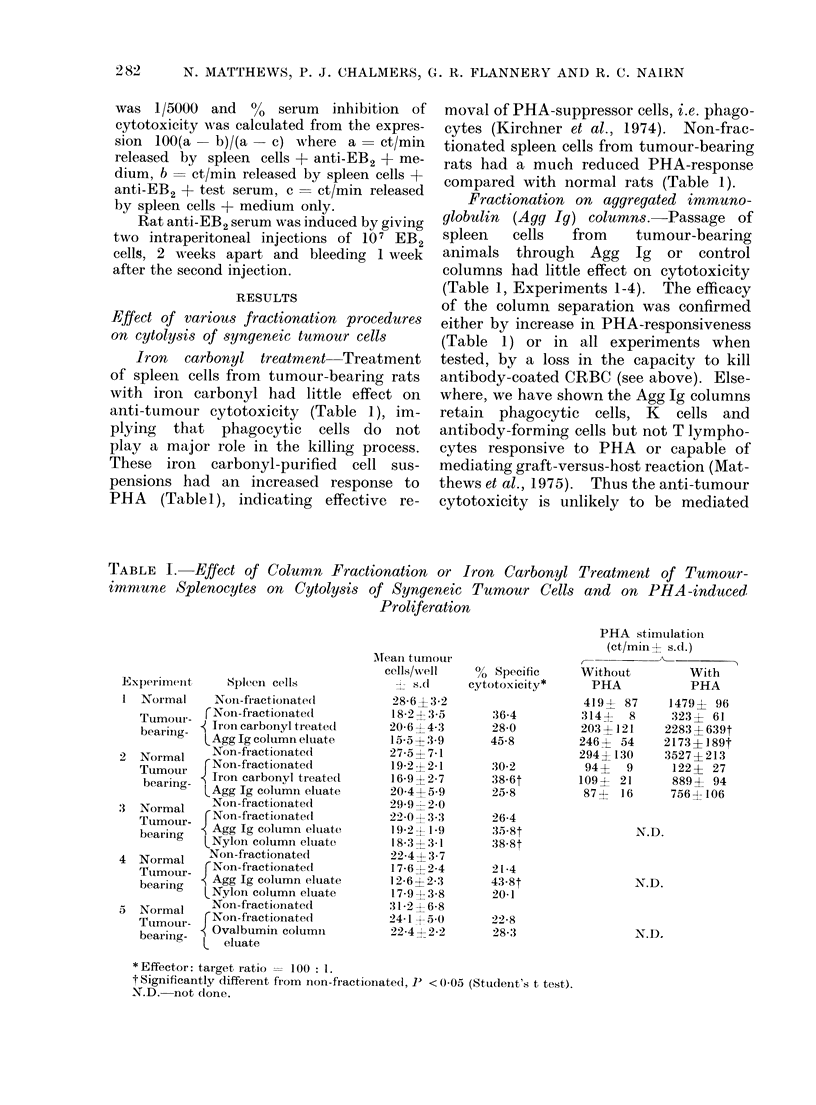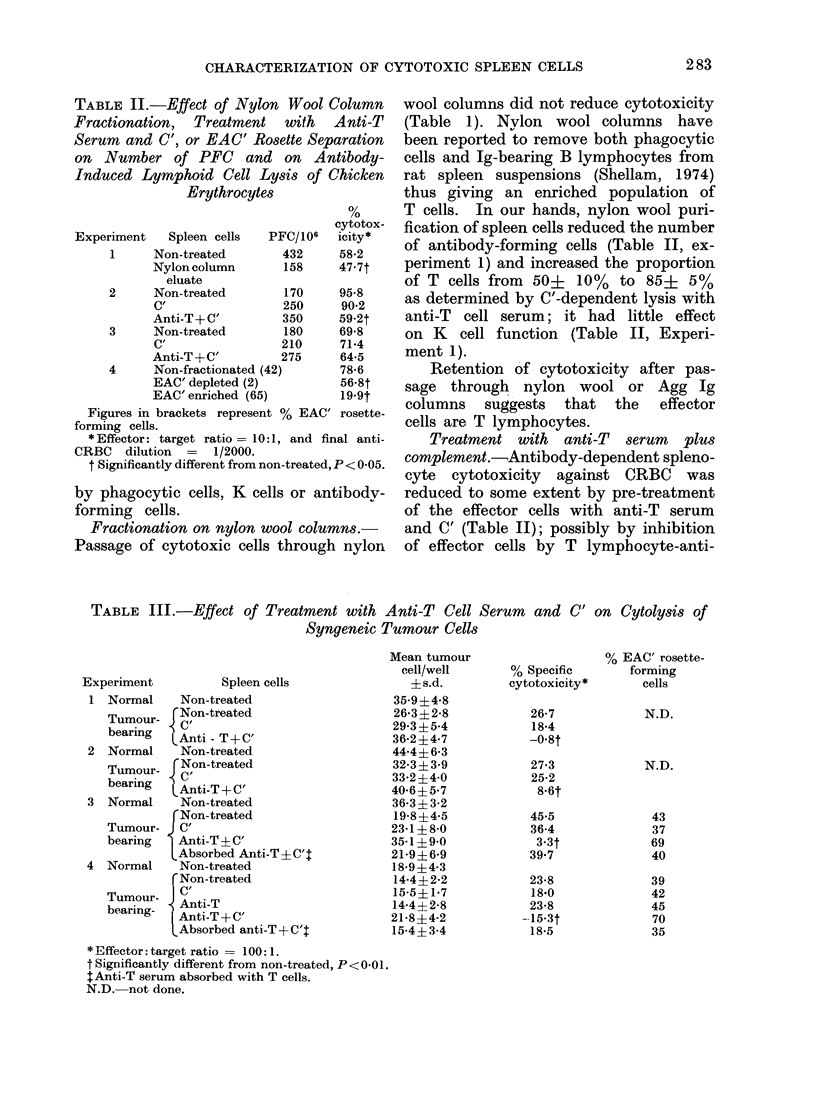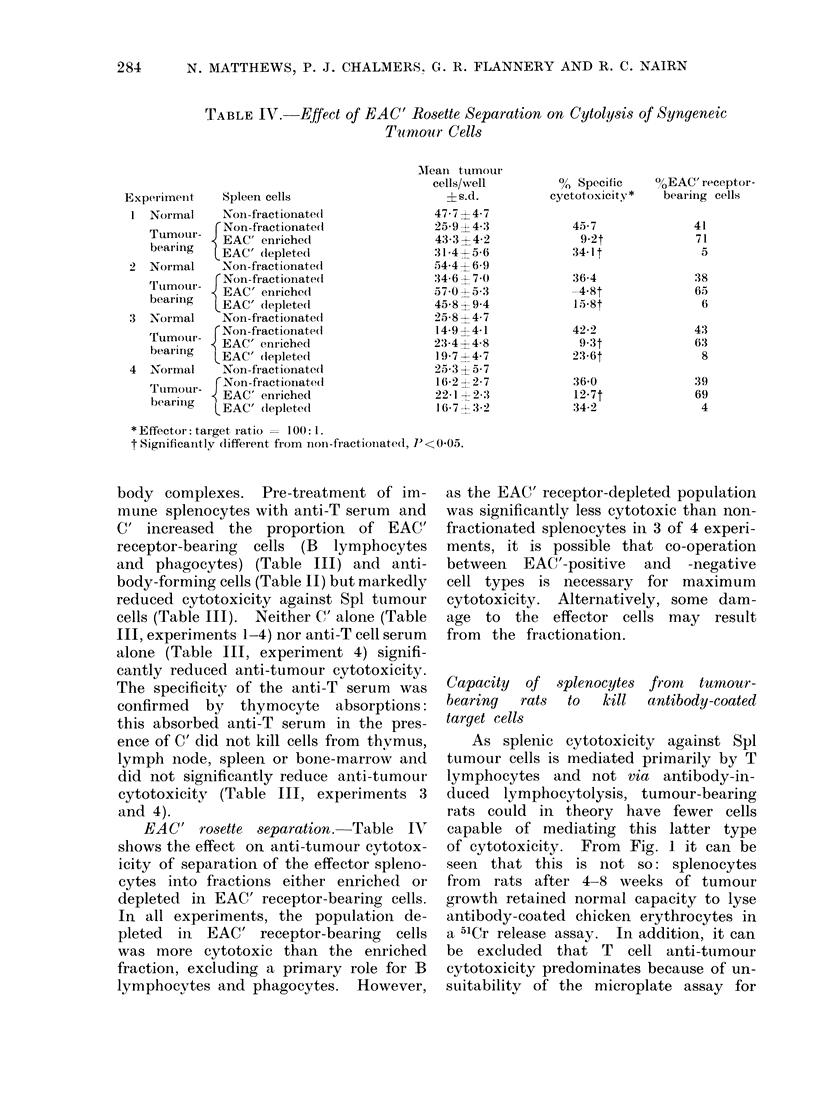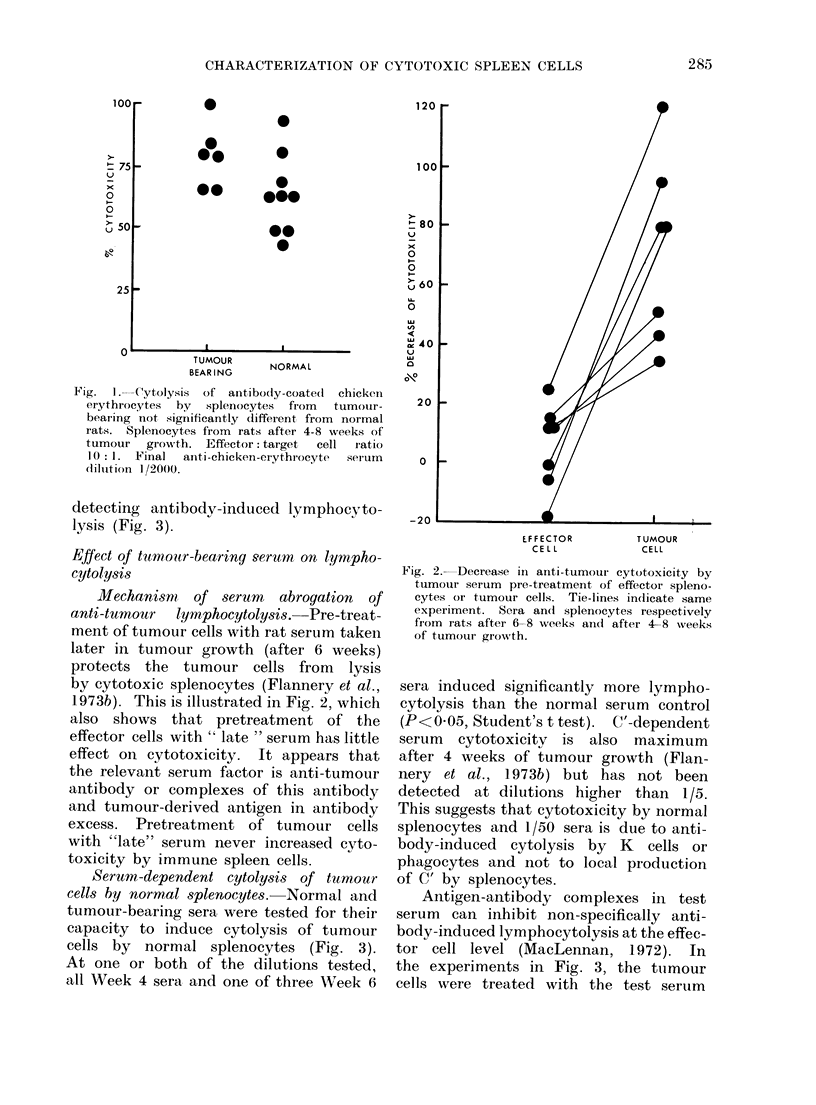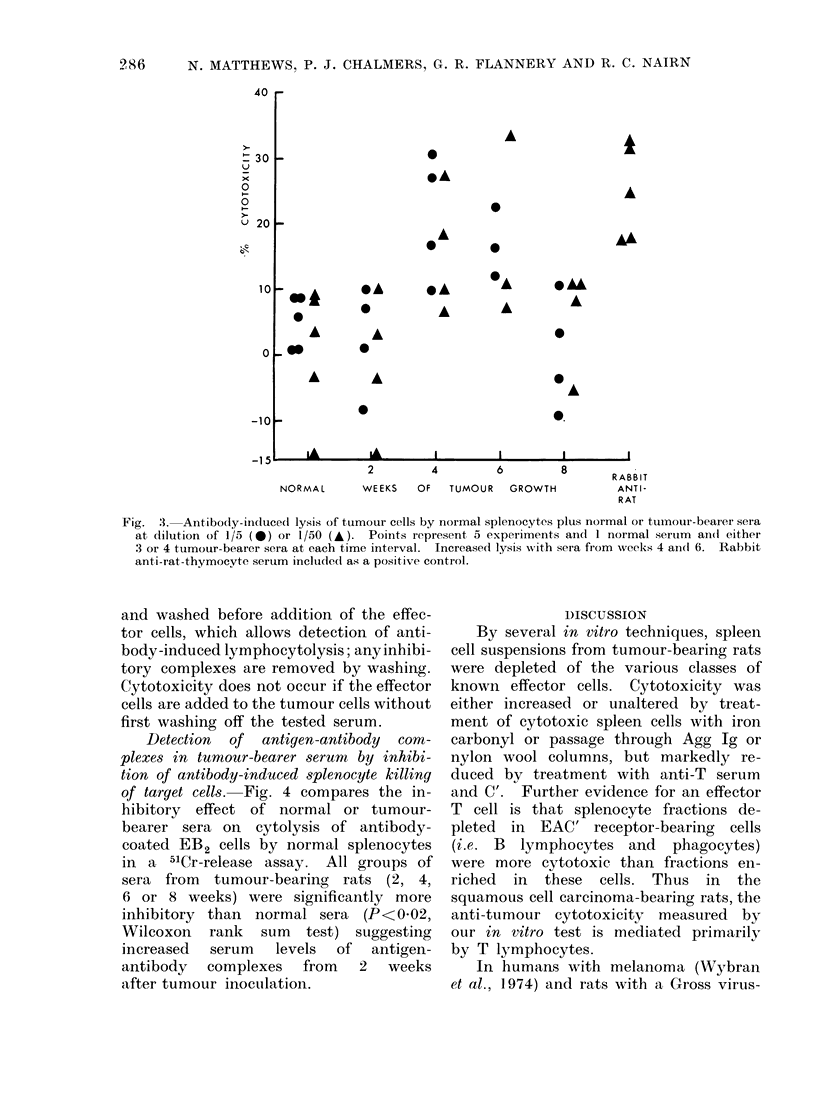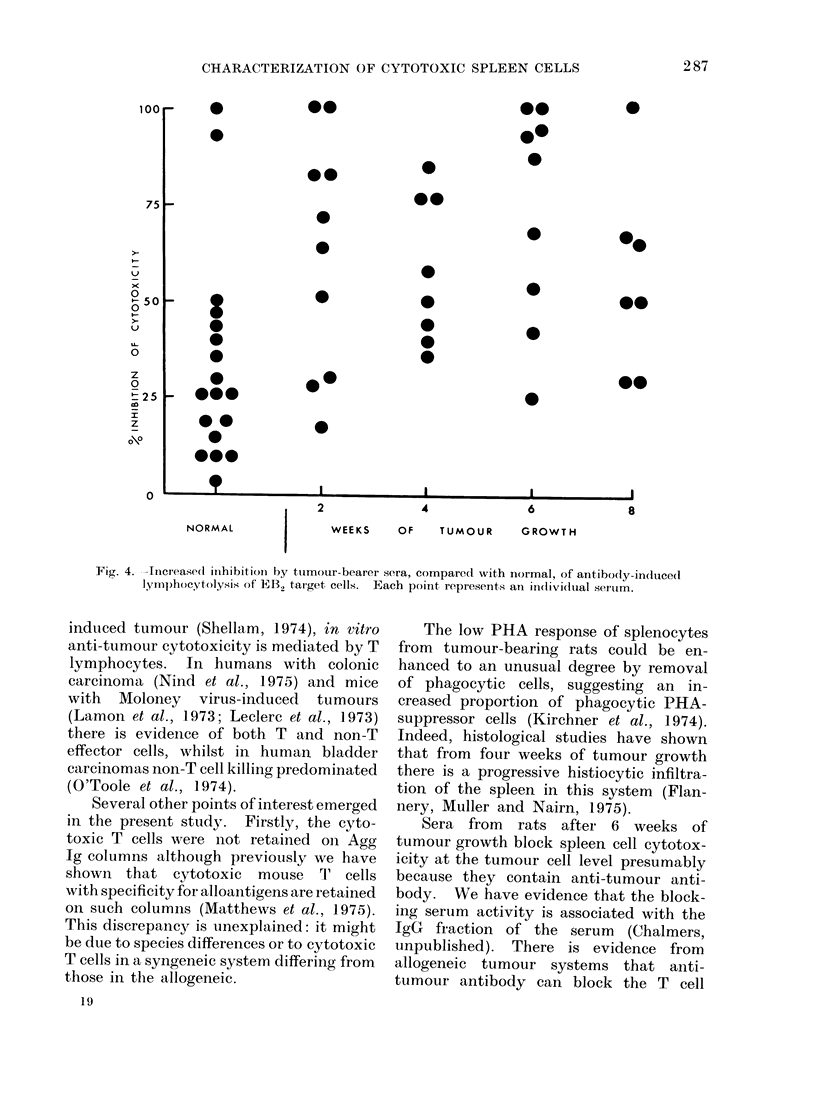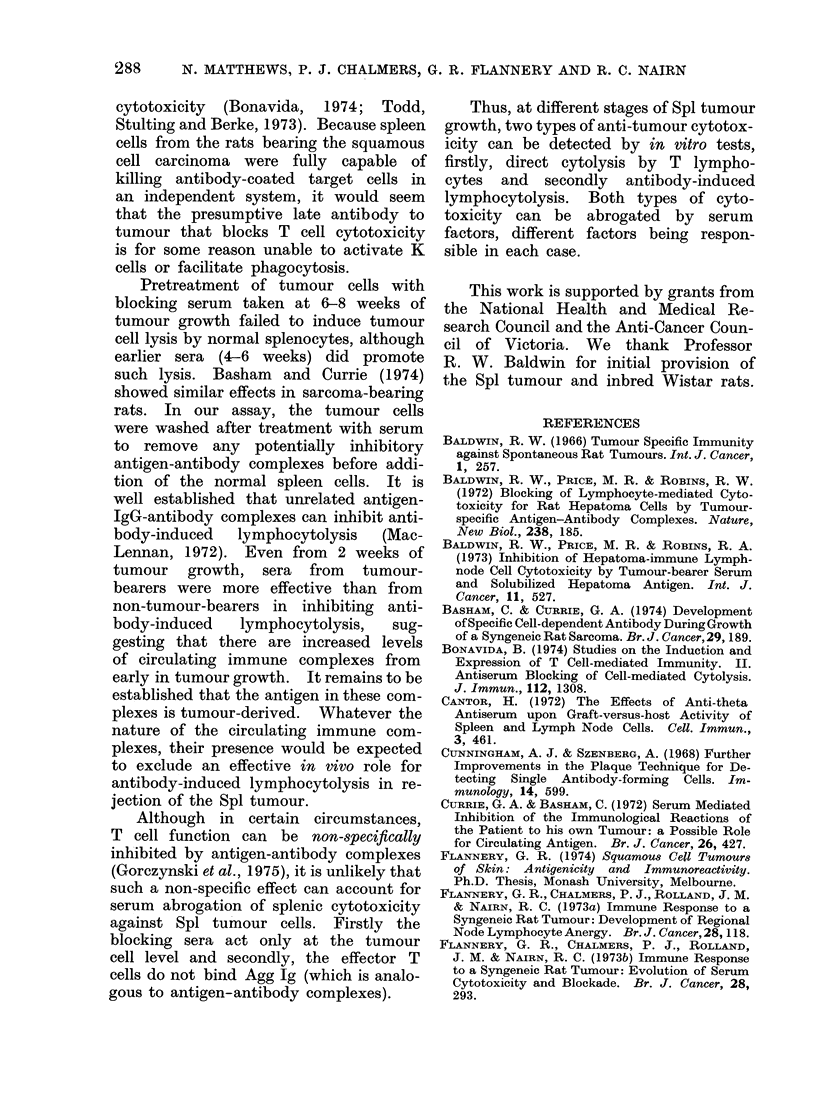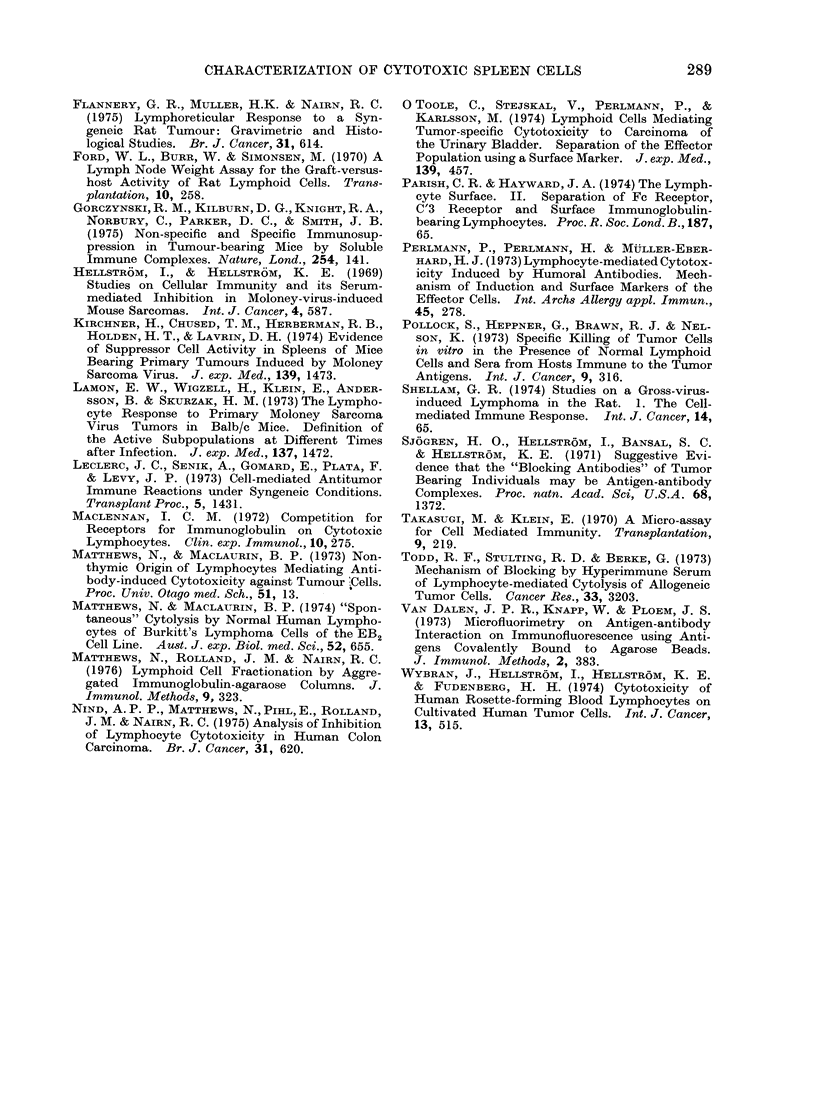# Characterization of cytotoxic spleen cells and effects of serum factors in a syngeneic rat tumour system.

**DOI:** 10.1038/bjc.1976.41

**Published:** 1976-03

**Authors:** N. Matthews, P. J. Chalmers, G. R. Flannery, R. C. Nairn

## Abstract

Splenocytes from inbred Wistar rats bearing a syngeneic squamous cell carcinoma (Spl) were fractionated by several techniques to characterize the lymphoid cells cytotoxic to the tumour in vitro. The anti-tumour cytotoxicity is presumably mediated primarily by T lymphocytes because it was greatly reduced by removal of T lymphocytes with heterologous anti-T serum plus complement but not by removal of other cell types. Cytotoxicity could be blocked at the tumour cell but not at the effector cell by sera taken late in tumour growth. Sera taken earlier in tumour growth could induce cytolysis of tumour cells by normal splenocytes but only if the tumour cells were treated with serum and washed before addition of the effector cells. Although splenocytes from normal and tumour-bearing rats were equally effective at lysing antibody-coated target cells it is unlikely that this mechanism is important in vivo as sera from early in tumour growth onwards contained factors (immune complexes?) which inhibited antibody-induced lymphocytolysis.


*Br. J. Cancer* (1976) **33**, 279

### CHARACTERIZATION OF CYTOTOXIC SPLEEN CELLS AND EFFECTS OF SERUM FACTORS IN A SYNGENEIC RAT TUMOUR SYSTEM

N. MATTHEWS, P. J. CHALMERS, G. R. FLANNERY AND R. C. NAIRN

*From the Department of Pathology and Immunology, Monash University, Melbourne, Australia*

Received 11 August 1975 Accepted 1 December 1975

**Summary.—Splenocytes from inbred Wistar rats bearing a syngeneic squamous cell carcinoma (Spl) were fractionated by several techniques to characterize the lymphoid cells cytotoxic to the tumour *in vitro*. The anti-tumour cytotoxicity is presumably mediated primarily by T lymphocytes because it was greatly reduced by removal of T lymphocytes with heterologous anti-T serum plus complement but not by removal of other cell types. Cytotoxicity could be blocked at the tumour cell but not at the effector cell by sera taken late in tumour growth. Sera taken earlier in tumour growth could induce cytolysis of tumour cells by normal splenocytes but only if the tumour cells were treated with serum and washed before addition of the effector cells. Although splenocytes from normal and tumour-bearing rats were equally effective at lysing antibody-coated target cells it is unlikely that this mechanism is important *in vivo* as sera from early in tumour growth onwards contained factors (immune complexes?) which inhibited antibody-induced lymphocytolysis.***

MANY humans and animals with progressing tumours have cytotoxic lymphoid cells for the tumour *in vitro* and this cytotoxicity can often be abrogated by the host's serum (Hellström and Hellström, 1969). The serum factors appear to be tumour-derived antigen, specific antibody, or complexes of tumour antigen and antibody and they may operate on either the lymphoid or the tumour cell (Hellström and Hellström, 1969; Sjögren *et al.*, 1971; Currie and Basham, 1972; Baldwin, Price and Robins, 1972, 1973). In other studies, sera from tumour-bearing animals have been shown to increase cytolysis of tumour cells by lymphoid cells (Pollack *et al.*, 1973; Basham and Currie, 1974).

A full understanding of the mechanism of serum modulation of lymphocytolysis requires knowledge not only of the serum factors but of the nature of the cytotoxic lymphoid cell. In this paper, we examine both the cytotoxic lymphoid cells and their modulation by serum factors in rats bearing a syngeneic squamous cell carcinoma.

### MATERIALS AND METHODS

*Rats and tumour.*—Inbred Wistar rats were inoculated subcutaneously in the medial aspect of the right thigh with $10^4$ syngeneic squamous cell carcinoma cells (Spl). This tumour is weakly immunogenic in the syngeneic host and has been shown to induce some transplantation resistance *in vivo* (Baldwin, 1966). We have shown previously that splenic cytotoxicity is detectable from 4 weeks after tumour inoculation until death of the animal after 8–9 weeks tumour growth (Flannery *et al.*, 1973*a*). In the local graft-versus-host test of T cell function described below, $F_1$ hybrids between this Wistar subline and DA (Agouti) rats were used.

* "Lymphocytolysis" is used in this communication to mean cytotoxicity by lymphoreticular cells. "Antibody-induced" is an abbreviation for any activation of such cells by antigen–antibody complexes.

*Cytotoxicity against syngeneic squamous cell carcinoma cells.*—Cytotoxicity tests were carried out in Falcon microtitre plates (No. 3034, Falcon Plastics Co.) using a modification (Flannery *et al.*, 1973*a*) of the procedures of Takasugi and Klein (1970). Spl tumour cells in Medium 199 + 20% foetal calf serum were incubated in the plates at 37°C overnight. Adherent cells were counted by phase contrast microscopy after washing, and wells containing 50–100 cells were used in all tests. Spleen cell suspensions were then added to give a final effector to target cell ratio of 100 : 1. Six to 10 replicates of each test and control well were used. Plates were incubated for a further 40–48 h at 37°C. Plates were washed, fixed with methanol and adherent cells counted by light microscopy. Cytotoxicity was calculated as 100(Nc—Nt)/Nc where Nc and Nt are respectively the mean number of surviving tumour cells in wells with spleen cells from either non-tumour-bearing or tumour-bearing rats. In fractionation experiments, cytotoxicity was expressed relative to non-fractionated control cells because preliminary studies showed that fractionation of control cells had little effect on the number of surviving tumour cells.

Spleen cells from Spl tumour-bearing rats were cytotoxic to Spl cells *in vitro* but not to (i) another Wistar rat tumour (Walker 256 carcinoma), (ii) a mammary tumour of this inbred line, (iii) normal lung histiocytes from syngeneic animals, when compared with control (non-immune) spleen cells (Flannery, 1974).

*Other assays of lymphoid cell function.*—Phytohaemagglutinin (PHA)-induced proliferation of spleen cells was as described by Matthews and Maclaurin (1973), antibody-induced cytolysis of chicken erythrocytes (CRBC) was assayed as described by Matthews and Maclaurin (1974), and direct plaque-forming cells (PFC) to sheep erythrocytes were assayed by the method of Cunningham and Szenberg (1968).

### *Cell fractionation techniques*

Fractionation of spleen cells on aggregated immunoglobulin (Agg Ig) columns on treatment with iron carbonyl are described elsewhere (Matthews, Rolland and Nairn, 1975). Briefly, aggregated immunoglobulin coupled to agarose was prepared by the method of van Dalen, Knapp and Ploem (1973). Agarose–Agg Ig slurry was poured into 5 ml syringes plugged with cotton wool, washed with sterile saline and incubated with saline plus 10% foetal calf serum for at least 1 h at room temperature before use.

Cell suspensions, $5 \times 10^6$ cells/ml bed volume in 10% of the bed volume, were allowed to flow into the columns which were then sealed and incubated vertically for 30 min. Non-adherent cells were eluted at 37°C at a flow rate of 10–20 ml/h. This treatment markedly reduced the activity of non-immune spleen cells in killing antibody-coated chicken erythrocytes (CRBC), a function associated with Fc-receptor-bearing cells (Perlmann, Perlmann and Müller-Eberhard, 1973). In a typical experiment (Matthews *et al.*, 1975), cytotoxicity of non-fractionated spleen cells ($44{\cdot}7 \pm 3{\cdot}5\%$) was reduced after passage through an Agg Ig column to $3{\cdot}0 \pm 2{\cdot}8\%$ (mean ± S.D.).

Phagocytic cells were removed from spleen cell suspensions by incubation of $10^8$ splenocytes with 0·3 g carbonyl iron (B.D.H.) for 1 h at 37°C with occasional shaking. Non-phagocytic cells were decanted, phagocytes being retained by a magnet. In three experiments this gave a mean reduction of 41% in the proportion of phagocytes as determined on Leishman's stained smears. Fractionation on nylon-wool columns was according to Shellam (1974), except that $10^7$ instead of $5 \times 10^7$ splenocytes were applied to columns containing 0·3 g nylon fibres.

(*a*) *Treatment with a heterologous anti-T cell serum.*—Two outbred rabbits weighing 2·5 kg received at weekly intervals, three intravenous injections of respectively, $2 \times 10^8$ rat thymocytes, $6 \times 10^7$ nylon-wool-purified lymph node cells, and finally $3 \times 10^8$ thymocytes. This regimen was adopted to raise antibodies against peripheral T lymphocytes as well as thymocytes. The rabbits were bled one week after the final injection, sera were pooled, heat inactivated and absorbed once with rat erythrocytes and thrice with bone marrow cells (anti-T serum), or for neutralization, twice with thymocytes and once with nylon-wool-purified lymph node cells.

To remove T lymphocytes from spleen cell suspensions, $10^7$ splenocytes in 0·9 ml Medium 199 (hereafter called "medium")

were incubated for 15 min at 37°C with 0·1 ml of a 1 in 10 dilution of anti-T cell serum. Guinea-pig complement (0·1 ml) was added and after a further 30 min incubation at 37°C, the cells were washed twice, counted, and resuspended at a concentration of Trypan Blue-excluding cells which gave an effector : target ratio of 100 : 1.

The specificity of the rabbit anti-rat T cell serum studies was confirmed by several criteria. The lytic effect of anti-T serum plus complement on lymphoid cell suspensions from various organs was proportional to their reported T cell content. A plateau level of killing was observed only with thymocytes, hence the anti-T cell serum was used at the lowest dilution (1/10) giving maximum lysis of thymocytes, *i.e.* 92%. Under these conditions the antiserum killed 50–60% of lymph node cells, 40–60% of splenocytes and less than 5% of bone marrow cells.

In addition, treatment of normal Wistar spleen cells with anti-T cell serum plus complement markedly reduced their ability to generate a graft-versus-host reaction, an accepted T lymphocyte function (Cantor, 1972), in the popliteal lymph node assay of Ford, Burr and Simonsen (1970). Briefly, $10^7$ parental spleen cells were injected into the hind footpads of $F_1$ hybrid rats and the popliteal node weights determined after 7 days. Contralateral footpads were injected with medium alone. Test lymph node weights (means of groups of $3 \pm$ S.D.) after cell treatment with anti-T cell serum plus complement were significantly lower ($20·7 \pm 3·9$ mg) than after treatment with antiserum alone, complement alone, normal serum plus complement or after no treatment ($32·6 \pm 10·4$ mg; $P < 0·01$) by the Mann–Whitney test.

(*b*) *EAC′ rosette separation.*—Rat splenocytes were initially purified by centrifugation over a modified Hypaque/ficoll mixture (Parish and Hayward, 1974) for 10 min at 1200 *g*; the cells at the interface were collected, washed and resuspended at $5 \times 10^6$ cells/ml in medium. The methods of coating sheep erythrocytes (E) with antibody (A) and complement (C′), and EAC′ rosette formation with rat splenocytes are described elsewhere (Matthews *et al.*, 1975). After rosette formation, the cell suspension was layered on to "modified" Hypaque/ficoll and centrifuged at 1200 *g* for 10 min. There were fewer EAC′ rosette-forming cells at the interface, whilst the pellet was enriched in EAC′-positive cells. This latter population was treated for 10 min at room temperature with isotonic $NH_4Cl$ solution to lyse the erythrocytes and all cell populations were washed twice, counted and resuspended at the appropriate concentration for cytotoxicity testing. The proportion of EAC′ rosette-forming cells in each of the fractions was then determined, no attempt being made to classify the rosette-forming cells as lymphocytes or other cell types.

*Serum reactivity*

(*a*) *Effect of tumour-bearer sera on cytolysis of tumour cells by immune splenocytes.*—For pretreatment of tumour cells, 10 μl medium or 1/5 diluted serum were incubated for 1 h at 37°C with previously plated Spl cells (50–100). After washing the microplates once, immune spleen cells were added at an effector : target ratio of 100 : 1 and the assay was continued as described previously (Flannery *et al.*, 1973*b*).

For pretreatment of effector cells, 250 μl of medium or 1/5 diluted serum were incubated for 1 h at 37°C with $2·5 \times 10^5$ immune splenocytes. After washing once, the effector cells were added at a ratio of 100 : 1 to untreated Spl tumour cells in microplates.

Results were expressed as the % reduction in cytotoxicity for test serum treatment relative to normal serum treatment.

(*b*) *Capacity of tumour-bearer sera to induce cytolysis of tumour cells by normal splenocytes.*—Plated Spl cells were incubated for 1 h at 37°C with 10 μl medium or with serum diluted 1/5 or 1/50. After washing, normal splenocytes were added to give an effector : target ratio of 100 : 1 and the assay was continued as usual. Cytotoxicity was expressed relative to control wells containing medium in place of serum.

(*c*) *Serum inhibition of antibody-induced lysis of $EB_2$ target cells by normal splenocytes.*—For the $^{51}Cr$ release assays (Matthews and Maclaurin, 1974), the effector spleen cells ($2 \times 10^6$) in 0·4 ml medium were incubated for 30 min at 37°C with 50 μl of the test serum or medium, before addition of the $^{51}Cr$-labelled $EB_2$ target cells ($2 \times 10^4$) plus anti-$EB_2$ serum in a volume of 50 μl. The final culture dilution of anti-$EB_2$ serum was 1/5000 and % serum inhibition of cytotoxicity was calculated from the expression 100(a — b)/(a — c) where a = ct/min released by spleen cells + anti-$EB_2$ + medium, $b$ = ct/min released by spleen cells + anti-$EB_2$ + test serum, c = ct/min released by spleen cells + medium only.

Rat anti-$EB_2$ serum was induced by giving two intraperitoneal injections of $10^7$ $EB_2$ cells, 2 weeks apart and bleeding 1 week after the second injection.

### RESULTS

### *Effect of various fractionation procedures on cytolysis of syngeneic tumour cells*

*Iron carbonyl treatment*—Treatment of spleen cells from tumour-bearing rats with iron carbonyl had little effect on anti-tumour cytotoxicity (Table 1), implying that phagocytic cells do not play a major role in the killing process. These iron carbonyl-purified cell suspensions had an increased response to PHA (Table1), indicating effective removal of PHA-suppressor cells, *i.e.* phagocytes (Kirchner *et al.*, 1974). Non-fractionated spleen cells from tumour-bearing rats had a much reduced PHA-response compared with normal rats (Table 1).

*Fractionation on aggregated immunoglobulin (Agg Ig) columns.*—Passage of spleen cells from tumour-bearing animals through Agg Ig or control columns had little effect on cytotoxicity (Table 1, Experiments 1-4). The efficacy of the column separation was confirmed either by increase in PHA-responsiveness (Table 1) or in all experiments when tested, by a loss in the capacity to kill antibody-coated CRBC (see above). Elsewhere, we have shown the Agg Ig columns retain phagocytic cells, K cells and antibody-forming cells but not T lymphocytes responsive to PHA or capable of mediating graft-versus-host reaction (Matthews *et al.*, 1975). Thus the anti-tumour cytotoxicity is unlikely to be mediated

TABLE I.—*Effect of Column Fractionation or Iron Carbonyl Treatment of Tumour-immune Splenocytes on Cytolysis of Syngeneic Tumour Cells and on PHA-induced Proliferation*

| Experiment | | Spleen cells | Mean tumour cells/well ± s.d | % Specific cytotoxicity* | PHA stimulation (ct/min ± s.d.) Without PHA | With PHA |
|---|---|---|---|---|---|---|
| 1 | Normal | Non-fractionated | 28·6±3·2 | | 419± 87 | 1479± 96 |
| | Tumour-bearing- | Non-fractionated | 18·2±3·5 | 36·4 | 314± 8 | 323± 61 |
| | | Iron carbonyl treated | 20·6±4·3 | 28·0 | 203±121 | 2283±639† |
| | | Agg Ig column eluate | 15·5±3·9 | 45·8 | 246± 54 | 2173±189† |
| 2 | Normal | Non-fractionated | 27·5±7·1 | | 294±130 | 3527±213 |
| | Tumour bearing- | Non-fractionated | 19·2±2·1 | 30·2 | 94± 9 | 122± 27 |
| | | Iron carbonyl treated | 16·9±2·7 | 38·6† | 109± 21 | 889± 94 |
| | | Agg Ig column eluate | 20·4±5·9 | 25·8 | 87± 16 | 756±106 |
| 3 | Normal | Non-fractionated | 29·9±2·0 | | | |
| | Tumour-bearing | Non-fractionated | 22·0±3·3 | 26·4 | N.D. | |
| | | Agg Ig column eluate | 19·2±1·9 | 35·8† | | |
| | | Nylon column eluate | 18·3±3·1 | 38·8† | | |
| 4 | Normal | Non-fractionated | 22·4±3·7 | | | |
| | Tumour-bearing | Non-fractionated | 17·6±2·4 | 21·4 | N.D. | |
| | | Agg Ig column eluate | 12·6±2·3 | 43·8† | | |
| | | Nylon column eluate | 17·9±3·8 | 20·1 | | |
| 5 | Normal | Non-fractionated | 31·2±6·8 | | | |
| | Tumour-bearing- | Non-fractionated | 24·1±5·0 | 22·8 | N.D. | |
| | | Ovalbumin column eluate | 22·4±2·2 | 28·3 | | |

*Effector: target ratio = 100 : 1.
†Significantly different from non-fractionated, $P < 0{\cdot}05$ (Student's t test).
N.D.—not done.

TABLE II.—*Effect of Nylon Wool Column Fractionation, Treatment with Anti-T Serum and C′, or EAC′ Rosette Separation on Number of PFC and on Antibody-Induced Lymphoid Cell Lysis of Chicken Erythrocytes*

| Experiment | Spleen cells | $PFC/10^6$ | % cytotoxicity* |
|---|---|---|---|
| 1 | Non-treated | 432 | 58·2 |
| | Nylon column eluate | 158 | 47·7† |
| 2 | Non-treated | 170 | 95·8 |
| | C′ | 250 | 90·2 |
| | Anti-T+C′ | 350 | 59·2† |
| 3 | Non-treated | 180 | 69·8 |
| | C′ | 210 | 71·4 |
| | Anti-T+C′ | 275 | 64·5 |
| 4 | Non-fractionated (42) | | 78·6 |
| | EAC′ depleted (2) | | 56·8† |
| | EAC′ enriched (65) | | 19·9† |

Figures in brackets represent % EAC′ rosette-forming cells.
*Effector: target ratio = 10:1, and final anti-CRBC dilution = 1/2000.
† Significantly different from non-treated, $P < 0·05$.

by phagocytic cells, K cells or antibody-forming cells.

*Fractionation on nylon wool columns.*—Passage of cytotoxic cells through nylon wool columns did not reduce cytotoxicity (Table 1). Nylon wool columns have been reported to remove both phagocytic cells and Ig-bearing B lymphocytes from rat spleen suspensions (Shellam, 1974) thus giving an enriched population of T cells. In our hands, nylon wool purification of spleen cells reduced the number of antibody-forming cells (Table II, experiment 1) and increased the proportion of T cells from 50± 10% to 85± 5% as determined by C′-dependent lysis with anti-T cell serum; it had little effect on K cell function (Table II, Experiment 1).

Retention of cytotoxicity after passage through nylon wool or Agg Ig columns suggests that the effector cells are T lymphocytes.

*Treatment with anti-T serum plus complement.*—Antibody-dependent splenocyte cytotoxicity against CRBC was reduced to some extent by pre-treatment of the effector cells with anti-T serum and C′ (Table II); possibly by inhibition of effector cells by T lymphocyte-anti-

TABLE III.—*Effect of Treatment with Anti-T Cell Serum and C′ on Cytolysis of Syngeneic Tumour Cells*

| Experiment | | Spleen cells | Mean tumour cell/well ±s.d. | % Specific cytotoxicity* | % EAC′ rosette-forming cells |
|---|---|---|---|---|---|
| 1 | Normal | Non-treated | 35·9±4·8 | | |
| | Tumour-bearing | Non-treated | 26·3±2·8 | 26·7 | N.D. |
| | | C′ | 29·3±5·4 | 18·4 | |
| | | Anti - T+C′ | 36·2±4·7 | −0·8† | |
| 2 | Normal | Non-treated | 44·4±6·3 | | |
| | Tumour-bearing | Non-treated | 32·3±3·9 | 27·3 | N.D. |
| | | C′ | 33·2±4·0 | 25·2 | |
| | | Anti-T+C′ | 40·6±5·7 | 8·6† | |
| 3 | Normal | Non-treated | 36·3±3·2 | | |
| | Tumour-bearing | Non-treated | 19·8±4·5 | 45·5 | 43 |
| | | C′ | 23·1±8·0 | 36·4 | 37 |
| | | Anti-T±C′ | 35·1±9·0 | 3·3† | 69 |
| | | Absorbed Anti-T±C′‡ | 21·9±6·9 | 39·7 | 40 |
| 4 | Normal | Non-treated | 18·9±4·3 | | |
| | Tumour-bearing- | Non-treated | 14·4±2·2 | 23·8 | 39 |
| | | C′ | 15·5±1·7 | 18·0 | 42 |
| | | Anti-T | 14·4±2·8 | 23·8 | 45 |
| | | Anti-T+C′ | 21·8±4·2 | −15·3† | 70 |
| | | Absorbed anti-T+C′‡ | 15·4±3·4 | 18·5 | 35 |

*Effector: target ratio = 100:1.
† Significantly different from non-treated, $P<0·01$.
‡Anti-T serum absorbed with T cells.
N.D.—not done.

TABLE IV.—*Effect of EAC′ Rosette Separation on Cytolysis of Syngeneic Tumour Cells*

| Experiment | | Spleen cells | Mean tumour cells/well ±s.d. | % Specific cyctotoxicity* | %EAC′ receptor-bearing cells |
|---|---|---|---|---|---|
| 1 | Normal | Non-fractionated | 47·7±4·7 | | |
| | Tumour-bearing | Non-fractionated | 25·9±4·3 | 45·7 | 41 |
| | | EAC′ enriched | 43·3±4·2 | 9·2† | 71 |
| | | EAC′ depleted | 31·4±5·6 | 34·1† | 5 |
| 2 | Normal | Non-fractionated | 54·4±6·9 | | |
| | Tumour-bearing | Non-fractionated | 34·6±7·0 | 36·4 | 38 |
| | | EAC′ enriched | 57·0±5·3 | −4·8† | 65 |
| | | EAC′ depleted | 45·8±9·4 | 15·8† | 6 |
| 3 | Normal | Non-fractionated | 25·8±4·7 | | |
| | Tumour-bearing | Non-fractionated | 14·9±4·1 | 42·2 | 43 |
| | | EAC′ enriched | 23·4±4·8 | 9·3† | 63 |
| | | EAC′ depleted | 19·7±4·7 | 23·6† | 8 |
| 4 | Normal | Non-fractionated | 25·3±5·7 | | |
| | Tumour-bearing | Non-fractionated | 16·2±2·7 | 36·0 | 39 |
| | | EAC′ enriched | 22·1±2·3 | 12·7† | 69 |
| | | EAC′ depleted | 16·7±3·2 | 34·2 | 4 |

*Effector: target ratio = 100:1.
† Significantly different from non-fractionated, $P<0·05$.

body complexes. Pre-treatment of immune splenocytes with anti-T serum and C′ increased the proportion of EAC′ receptor-bearing cells (B lymphocytes and phagocytes) (Table III) and antibody-forming cells (Table II) but markedly reduced cytotoxicity against Spl tumour cells (Table III). Neither C′ alone (Table III, experiments 1–4) nor anti-T cell serum alone (Table III, experiment 4) significantly reduced anti-tumour cytotoxicity. The specificity of the anti-T serum was confirmed by thymocyte absorptions: this absorbed anti-T serum in the presence of C′ did not kill cells from thymus, lymph node, spleen or bone-marrow and did not significantly reduce anti-tumour cytotoxicity (Table III, experiments 3 and 4).

*EAC′ rosette separation.*—Table IV shows the effect on anti-tumour cytotoxicity of separation of the effector splenocytes into fractions either enriched or depleted in EAC′ receptor-bearing cells. In all experiments, the population depleted in EAC′ receptor-bearing cells was more cytotoxic than the enriched fraction, excluding a primary role for B lymphocytes and phagocytes. However, as the EAC′ receptor-depleted population was significantly less cytotoxic than non-fractionated splenocytes in 3 of 4 experiments, it is possible that co-operation between EAC′-positive and -negative cell types is necessary for maximum cytotoxicity. Alternatively, some damage to the effector cells may result from the fractionation.

*Capacity of splenocytes from tumour-bearing rats to kill antibody-coated target cells*

As splenic cytotoxicity against Spl tumour cells is mediated primarily by T lymphocytes and not *via* antibody-induced lymphocytolysis, tumour-bearing rats could in theory have fewer cells capable of mediating this latter type of cytotoxicity. From Fig. 1 it can be seen that this is not so: splenocytes from rats after 4–8 weeks of tumour growth retained normal capacity to lyse antibody-coated chicken erythrocytes in a $^{51}Cr$ release assay. In addition, it can be excluded that T cell anti-tumour cytotoxicity predominates because of unsuitability of the microplate assay for

Fig. 1.—Cytolysis of antibody-coated chicken erythrocytes by splenocytes from tumour-bearing not significantly different from normal rats. Splenocytes from rats after 4-8 weeks of tumour growth. Effector : target cell ratio 10 : 1. Final anti-chicken-erythrocyte serum dilution 1/2000.

Fig. 2.—Decrease in anti-tumour cytotoxicity by tumour serum pre-treatment of effector splenocytes or tumour cells. Tie-lines indicate same experiment. Sera and splenocytes respectively from rats after 6–8 weeks and after 4–8 weeks of tumour growth.

detecting antibody-induced lymphocytolysis (Fig. 3).

*Effect of tumour-bearing serum on lymphocytolysis*

*Mechanism of serum abrogation of anti-tumour lymphocytolysis*.—Pre-treatment of tumour cells with rat serum taken later in tumour growth (after 6 weeks) protects the tumour cells from lysis by cytotoxic splenocytes (Flannery *et al.*, 1973*b*). This is illustrated in Fig. 2, which also shows that pretreatment of the effector cells with " late " serum has little effect on cytotoxicity. It appears that the relevant serum factor is anti-tumour antibody or complexes of this antibody and tumour-derived antigen in antibody excess. Pretreatment of tumour cells with "late" serum never increased cytotoxicity by immune spleen cells.

*Serum-dependent cytolysis of tumour cells by normal splenocytes*.—Normal and tumour-bearing sera were tested for their capacity to induce cytolysis of tumour cells by normal splenocytes (Fig. 3). At one or both of the dilutions tested, all Week 4 sera and one of three Week 6 sera induced significantly more lymphocytolysis than the normal serum control ($P<0{\cdot}05$, Student's t test). C′-dependent serum cytotoxicity is also maximum after 4 weeks of tumour growth (Flannery *et al.*, 1973*b*) but has not been detected at dilutions higher than 1/5. This suggests that cytotoxicity by normal splenocytes and 1/50 sera is due to antibody-induced cytolysis by K cells or phagocytes and not to local production of C′ by splenocytes.

Antigen-antibody complexes in test serum can inhibit non-specifically antibody-induced lymphocytolysis at the effector cell level (MacLennan, 1972). In the experiments in Fig. 3, the tumour cells were treated with the test serum

Fig. 3.—Antibody-induced lysis of tumour cells by normal splenocytes plus normal or tumour-bearer sera at dilution of 1/5 (●) or 1/50 (▲). Points represent 5 experiments and 1 normal serum and either 3 or 4 tumour-bearer sera at each time interval. Increased lysis with sera from weeks 4 and 6. Rabbit anti-rat-thymocyte serum included as a positive control.

and washed before addition of the effector cells, which allows detection of antibody-induced lymphocytolysis; any inhibitory complexes are removed by washing. Cytotoxicity does not occur if the effector cells are added to the tumour cells without first washing off the tested serum.

*Detection of antigen-antibody complexes in tumour-bearer serum by inhibition of antibody-induced splenocyte killing of target cells.*—Fig. 4 compares the inhibitory effect of normal or tumour-bearer sera on cytolysis of antibody-coated $EB_2$ cells by normal splenocytes in a $^{51}$Cr-release assay. All groups of sera from tumour-bearing rats (2, 4, 6 or 8 weeks) were significantly more inhibitory than normal sera ($P<0{\cdot}02$, Wilcoxon rank sum test) suggesting increased serum levels of antigen-antibody complexes from 2 weeks after tumour inoculation.

### DISCUSSION

By several *in vitro* techniques, spleen cell suspensions from tumour-bearing rats were depleted of the various classes of known effector cells. Cytotoxicity was either increased or unaltered by treatment of cytotoxic spleen cells with iron carbonyl or passage through Agg Ig or nylon wool columns, but markedly reduced by treatment with anti-T serum and C′. Further evidence for an effector T cell is that splenocyte fractions depleted in EAC′ receptor-bearing cells (*i.e.* B lymphocytes and phagocytes) were more cytotoxic than fractions enriched in these cells. Thus in the squamous cell carcinoma-bearing rats, the anti-tumour cytotoxicity measured by our *in vitro* test is mediated primarily by T lymphocytes.

In humans with melanoma (Wybran *et al.*, 1974) and rats with a Gross virus-

Fig. 4.—Increased inhibition by tumour-bearer sera, compared with normal, of antibody-induced lymphocytolysis of $EB_2$ target cells. Each point represents an individual serum.

induced tumour (Shellam, 1974), *in vitro* anti-tumour cytotoxicity is mediated by T lymphocytes. In humans with colonic carcinoma (Nind *et al.*, 1975) and mice with Moloney virus-induced tumours (Lamon *et al.*, 1973; Leclerc *et al.*, 1973) there is evidence of both T and non-T effector cells, whilst in human bladder carcinomas non-T cell killing predominated (O'Toole *et al.*, 1974).

Several other points of interest emerged in the present study. Firstly, the cytotoxic T cells were not retained on Agg Ig columns although previously we have shown that cytotoxic mouse T cells with specificity for alloantigens are retained on such columns (Matthews *et al.*, 1975). This discrepancy is unexplained: it might be due to species differences or to cytotoxic T cells in a syngeneic system differing from those in the allogeneic.

The low PHA response of splenocytes from tumour-bearing rats could be enhanced to an unusual degree by removal of phagocytic cells, suggesting an increased proportion of phagocytic PHA-suppressor cells (Kirchner *et al.*, 1974). Indeed, histological studies have shown that from four weeks of tumour growth there is a progressive histiocytic infiltration of the spleen in this system (Flannery, Muller and Nairn, 1975).

Sera from rats after 6 weeks of tumour growth block spleen cell cytotoxicity at the tumour cell level presumably because they contain anti-tumour antibody. We have evidence that the blocking serum activity is associated with the IgG fraction of the serum (Chalmers, unpublished). There is evidence from allogeneic tumour systems that anti-tumour antibody can block the T cell cytotoxicity (Bonavida, 1974; Todd, Stulting and Berke, 1973). Because spleen cells from the rats bearing the squamous cell carcinoma were fully capable of killing antibody-coated target cells in an independent system, it would seem that the presumptive late antibody to tumour that blocks T cell cytotoxicity is for some reason unable to activate K cells or facilitate phagocytosis.

Pretreatment of tumour cells with blocking serum taken at 6–8 weeks of tumour growth failed to induce tumour cell lysis by normal splenocytes, although earlier sera (4–6 weeks) did promote such lysis. Basham and Currie (1974) showed similar effects in sarcoma-bearing rats. In our assay, the tumour cells were washed after treatment with serum to remove any potentially inhibitory antigen-antibody complexes before addition of the normal spleen cells. It is well established that unrelated antigen-IgG-antibody complexes can inhibit antibody-induced lymphocytolysis (MacLennan, 1972). Even from 2 weeks of tumour growth, sera from tumour-bearers were more effective than from non-tumour-bearers in inhibiting antibody-induced lymphocytolysis, suggesting that there are increased levels of circulating immune complexes from early in tumour growth. It remains to be established that the antigen in these complexes is tumour-derived. Whatever the nature of the circulating immune complexes, their presence would be expected to exclude an effective *in vivo* role for antibody-induced lymphocytolysis in rejection of the Spl tumour.

Although in certain circumstances, T cell function can be *non-specifically* inhibited by antigen-antibody complexes (Gorczynski *et al.*, 1975), it is unlikely that such a non-specific effect can account for serum abrogation of splenic cytotoxicity against Spl tumour cells. Firstly the blocking sera act only at the tumour cell level and secondly, the effector T cells do not bind Agg Ig (which is analogous to antigen–antibody complexes).

Thus, at different stages of Spl tumour growth, two types of anti-tumour cytotoxicity can be detected by *in vitro* tests, firstly, direct cytolysis by T lymphocytes and secondly antibody-induced lymphocytolysis. Both types of cytotoxicity can be abrogated by serum factors, different factors being responsible in each case.

This work is supported by grants from the National Health and Medical Research Council and the Anti-Cancer Council of Victoria. We thank Professor R. W. Baldwin for initial provision of the Spl tumour and inbred Wistar rats.

### REFERENCES

BALDWIN, R. W. (1966) Tumour Specific Immunity against Spontaneous Rat Tumours. *Int. J. Cancer*, **1**, 257.

BALDWIN, R. W., PRICE, M. R. & ROBINS, R. W. (1972) Blocking of Lymphocyte-mediated Cytotoxicity for Rat Hepatoma Cells by Tumour-specific Antigen–Antibody Complexes. *Nature, New Biol.*, **238**, 185.

BALDWIN, R. W., PRICE, M. R. & ROBINS, R. A. (1973) Inhibition of Hepatoma-immune Lymph-node Cell Cytotoxicity by Tumour-bearer Serum and Solubilized Hepatoma Antigen. *Int. J. Cancer*, **11**, 527.

BASHAM, C. & CURRIE, G. A. (1974) Development of Specific Cell-dependent Antibody During Growth of a Syngeneic Rat Sarcoma. *Br. J. Cancer*, **29**, 189.

BONAVIDA, B. (1974) Studies on the Induction and Expression of T Cell-mediated Immunity. II. Antiserum Blocking of Cell-mediated Cytolysis. *J. Immun.*, **112**, 1308.

CANTOR, H. (1972) The Effects of Anti-theta Antiserum upon Graft-versus-host Activity of Spleen and Lymph Node Cells. *Cell. Immun.*, **3**, 461.

CUNNINGHAM, A. J. & SZENBERG, A. (1968) Further Improvements in the Plaque Technique for Detecting Single Antibody-forming Cells. *Immunology*, **14**, 599.

CURRIE, G. A. & BASHAM, C. (1972) Serum Mediated Inhibition of the Immunological Reactions of the Patient to his own Tumour: a Possible Role for Circulating Antigen. *Br. J. Cancer*, **26**, 427.

FLANNERY, G. R. (1974) *Squamous Cell Tumours of Skin: Antigenicity and Immunoreactivity.* Ph.D. Thesis, Monash University, Melbourne.

FLANNERY, G. R., CHALMERS, P. J., ROLLAND, J. M. & NAIRN, R. C. (1973*a*) Immune Response to a Syngeneic Rat Tumour: Development of Regional Node Lymphocyte Anergy. *Br. J. Cancer*, **28**, 118.

FLANNERY, G. R., CHALMERS, P. J., ROLLAND, J. M. & NAIRN, R. C. (1973*b*) Immune Response to a Syngeneic Rat Tumour: Evolution of Serum Cytotoxicity and Blockade. *Br. J. Cancer*, **28**, 293.

FLANNERY, G. R., MULLER, H.K. & NAIRN, R. C. (1975) Lymphoreticular Response to a Syngeneic Rat Tumour: Gravimetric and Histological Studies. *Br. J. Cancer*, **31**, 614.

FORD, W. L., BURR, W. & SIMONSEN, M. (1970) A Lymph Node Weight Assay for the Graft-versus-host Activity of Rat Lymphoid Cells. *Transplantation*, **10**, 258.

GORCZYNSKI, R. M., KILBURN, D. G., KNIGHT, R. A., NORBURY, C., PARKER, D. C., & SMITH, J. B. (1975) Non-specific and Specific Immunosuppression in Tumour-bearing Mice by Soluble Immune Complexes. *Nature, Lond.*, **254**, 141.

HELLSTRÖM, I., & HELLSTRÖM, K. E. (1969) Studies on Cellular Immunity and its Serum-mediated Inhibition in Moloney-virus-induced Mouse Sarcomas. *Int. J. Cancer*, **4**, 587.

KIRCHNER, H., CHUSED, T. M., HERBERMAN, R. B., HOLDEN, H. T., & LAVRIN, D. H. (1974) Evidence of Suppressor Cell Activity in Spleens of Mice Bearing Primary Tumours Induced by Moloney Sarcoma Virus. *J. exp. Med.*, **139**, 1473.

LAMON, E. W., WIGZELL, H., KLEIN, E., ANDERSSON, B. & SKURZAK, H. M. (1973) The Lymphocyte Response to Primary Moloney Sarcoma Virus Tumors in Balb/c Mice. Definition of the Active Subpopulations at Different Times after Infection. *J. exp. Med.*, **137**, 1472.

LECLERC, J. C., SENIK, A., GOMARD, E., PLATA, F. & LEVY, J. P. (1973) Cell-mediated Antitumor Immune Reactions under Syngeneic Conditions. *Transplant Proc.*, **5**, 1431.

MACLENNAN, I. C. M. (1972) Competition for Receptors for Immunoglobulin on Cytotoxic Lymphocytes. *Clin. exp. Immunol.*, **10**, 275.

MATTHEWS, N., & MACLAURIN, B. P. (1973) Non-thymic Origin of Lymphocytes Mediating Antibody-induced Cytotoxicity against Tumour Cells. *Proc. Univ. Otago med. Sch.*, **51**, 13.

MATTHEWS, N. & MACLAURIN, B. P. (1974) "Spontaneous" Cytolysis by Normal Human Lymphocytes of Burkitt's Lymphoma Cells of the $EB_2$ Cell Line. *Aust. J. exp. Biol. med. Sci.*, **52**, 655.

MATTHEWS, N., ROLLAND, J. M. & NAIRN, R. C. (1976) Lymphoid Cell Fractionation by Aggregated Immunoglobulin-agaraose Columns. *J. Immunol. Methods*, **9**, 323.

NIND, A. P. P., MATTHEWS, N., PIHL, E., ROLLAND, J. M. & NAIRN, R. C. (1975) Analysis of Inhibition of Lymphocyte Cytotoxicity in Human Colon Carcinoma. *Br. J. Cancer*, **31**, 620.

O TOOLE, C., STEJSKAL, V., PERLMANN, P., & KARLSSON, M. (1974) Lymphoid Cells Mediating Tumor-specific Cytotoxicity to Carcinoma of the Urinary Bladder. Separation of the Effector Population using a Surface Marker. *J. exp. Med.*, **139**, 457.

PARISH, C. R. & HAYWARD, J. A. (1974) The Lymphcyte Surface. II. Separation of Fc Receptor, C'3 Receptor and Surface Immunoglobulin-bearing Lymphocytes. *Proc. R. Soc. Lond. B.*, **187**, 65.

PERLMANN, P., PERLMANN, H. & MÜLLER-EBERHARD, H. J. (1973) Lymphocyte-mediated Cytotoxicity Induced by Humoral Antibodies. Mechanism of Induction and Surface Markers of the Effector Cells. *Int. Archs Allergy appl. Immun.*, **45**, 278.

POLLOCK, S., HEPPNER, G., BRAWN, R. J. & NELSON, K. (1973) Specific Killing of Tumor Cells *in vitro* in the Presence of Normal Lymphoid Cells and Sera from Hosts Immune to the Tumor Antigens. *Int. J. Cancer*, **9**, 316.

SHELLAM, G. R. (1974) Studies on a Gross-virus-induced Lymphoma in the Rat. 1. The Cell-mediated Immune Response. *Int. J. Cancer*, **14**, 65.

SJÖGREN, H. O., HELLSTRÖM, I., BANSAL, S. C. & HELLSTRÖM, K. E. (1971) Suggestive Evidence that the "Blocking Antibodies" of Tumor Bearing Individuals may be Antigen-antibody Complexes. *Proc. natn. Acad. Sci, U.S.A.* **68**, 1372.

TAKASUGI, M. & KLEIN, E. (1970) A Micro-assay for Cell Mediated Immunity. *Transplantation*, **9**, 219.

TODD, R. F., STULTING, R. D. & BERKE, G. (1973) Mechanism of Blocking by Hyperimmune Serum of Lymphocyte-mediated Cytolysis of Allogeneic Tumor Cells. *Cancer Res.*, **33**, 3203.

VAN DALEN, J. P. R., KNAPP, W. & PLOEM, J. S. (1973) Microfluorimetry on Antigen-antibody Interaction on Immunofluorescence using Antigens Covalently Bound to Agarose Beads. *J. Immunol. Methods*, **2**, 383.

WYBRAN, J., HELLSTRÖM, I., HELLSTRÖM, K. E. & FUDENBERG, H. H. (1974) Cytotoxicity of Human Rosette-forming Blood Lymphocytes on Cultivated Human Tumor Cells. *Int. J. Cancer*, **13**, 515.